# Delayed-Interval Delivery in Multifetal In Vitro Fertilization (IVF) Pregnancies: Two Case Reports

**DOI:** 10.7759/cureus.81925

**Published:** 2025-04-08

**Authors:** Raluca Tocariu, Raluca F Mitroi, Lucia E Niculae, Ciprian A Coroleuca, Alexandru Blidaru

**Affiliations:** 1 Department of Obstetrics and Gynecology, Carol Davila University of Medicine and Pharmacy, Bucharest, ROU; 2 Department of Neonatology, Clinical Hospital of Obstetrics and Gynecology “Prof. Dr. Panait Sârbu”, Bucharest, ROU; 3 Department of Obstetrics and Gynecology, Clinical Hospital of Obstetrics and Gynecology “Prof. Dr. Panait Sârbu”, Bucharest, ROU; 4 Department of Surgery, Carol Davila University of Medicine and Pharmacy, Bucharest, ROU; 5 Department of Surgical Oncology, Institute of Oncology "Prof. Dr. Alexandru Trestioreanu", Bucharest, ROU

**Keywords:** delayed interval delivery, in vitro fertilization, neonatal outcomes, prematurity, preterm birth, twin pregnancy

## Abstract

Delayed-interval delivery in twin pregnancies is a rare but increasingly recognized obstetric intervention aimed at improving neonatal outcomes, particularly in cases of preterm labor. The rise in multiple gestations associated with in-vitro fertilization (IVF) has contributed to the need for optimized management strategies in such cases. However, there remains no consensus on standardized protocols for delayed twin delivery.

We present two cases of dichorionic-diamniotic twin pregnancies obtained via IVF, where the birth of the second twin was delayed by 5 and 30 days. In the first case, a 31-year-old nulliparous woman presented at 24 weeks of gestation with preterm labor. Despite initial tocolysis and antibiotic prophylaxis, the first twin was delivered at 24 weeks, while the second twin was successfully retained until 28 weeks. The first neonate developed severe retinopathy of prematurity, while the second twin exhibited normal neurodevelopmental outcomes. In the second case, a 52-year-old multiparous woman with an IVF pregnancy and donor oocytes experienced premature rupture of membranes at 25 weeks, leading to immediate vaginal delivery of the first twin. Conservative management, including tocolysis and antibiotics, enabled the extension of the pregnancy for five additional days. The first neonate had a favorable postnatal course, while the second twin developed posthemorrhagic hydrocephalus requiring ventriculoperitoneal shunting.

The decision to delay twin delivery is contingent upon multiple factors, including maternal stability, absence of chorioamnionitis, and the viability of the remaining fetus. Current literature suggests that prolonged latency between twin deliveries is associated with reduced neonatal morbidity when managed with strict maternal monitoring, tocolysis, and infection control. However, the benefits must be weighed against maternal and fetal risks, particularly the potential for intrauterine infection and placental dysfunction.

Asynchronous twin delivery remains a complex but potentially beneficial intervention for improving neonatal outcomes in cases of preterm labor, especially in IVF pregnancies. These cases highlight the importance of individualized management, vigilant maternal-fetal surveillance, and further research to establish standardized protocols for optimizing perinatal outcomes.

## Introduction

Multiple pregnancies constitute approximately 1% of all gestations, with this proportion increasing due to the rising use of assisted reproductive technologies (ART) [1,2]. The prevalence of multifetal pregnancies has escalated alongside advancements in reproductive medicine, leading to an increased risk of obstetric complications, most notably preterm labor and preterm birth. Also, compared to singleton pregnancies, twins and higher-order multiples are at an inherently elevated risk of complications such as respiratory distress syndrome, necrotizing enterocolitis, intraventricular hemorrhage, and long-term neurodevelopmental impairment [3-5].

In twin pregnancies, the delivery of the second twin traditionally follows shortly after the birth of the first fetus due to ongoing uterine contractions and placental detachment. However, in select cases, the preterm birth of one twin does not necessarily mandate the immediate delivery of the second fetus. With appropriate medical management, the remaining twin can be retained in utero, sometimes for an extended period, allowing for further fetal growth and maturation. Delayed-interval delivery, though relatively uncommon, is a strategic obstetric approach aimed at prolonging gestation and improving neonatal outcomes by reducing the risks associated with extreme prematurity [6].

Despite its potential benefits, the literature reveals a lack of consensus on the best approach for managing delayed twin delivery. Currently, there is no standardized protocol for optimizing maternal and fetal outcomes in cases of asynchronous twin births [7]. Several factors influence the feasibility of delayed delivery, including maternal stability, the presence or absence of chorioamnionitis, cervical competence, uterine contractility, and the condition of the remaining fetus. While tocolytic agents, antibiotics, and corticosteroids are commonly used to prolong pregnancy, their efficacy, duration of use, and potential complications remain subjects of ongoing debate [1,8].

This paper aims to contribute our clinical experience to the existing limited body of literature concerning the management of delayed twin delivery. Understanding the factors that influence latency duration, maternal-fetal risks, and neonatal outcomes is critical in refining management strategies for these complex pregnancies. Here, we present two cases of dichorionic-diamniotic twin pregnancies in which the interval between deliveries ranged from 5 to 30 days, providing insight into the challenges and benefits of delayed delivery. Through these cases, we highlight the importance of individualized management, stringent maternal-fetal surveillance, and further research to establish evidence-based guidelines for delayed-interval twin delivery.

## Case presentation

First case

A 31-year-old nulliparous woman with no significant medical or obstetric history presented at 24 weeks of gestation with a dichorionic, diamniotic twin pregnancy, following in vitro fertilization (IVF). The pregnancy was achieved through a successful embryo transfer of two blastocysts following an assisted reproductive technology cycle. Due to cervical incompetence, a cervical cerclage was placed at 18 weeks of gestation in an attempt to mitigate the risk of preterm birth.

Upon admission to the emergency department, a pelvic examination revealed bulging amniotic membranes through the cervical cerclage suture. Laboratory investigations demonstrated leukocytosis and elevated inflammatory markers, indicative of chorioamnionitis. Despite these findings, the amniotic membranes remained intact, and ultrasonography confirmed the presence of two viable fetuses, weighing 661 g and 793 g, respectively.

Tocolysis was initiated with hexoprenaline, a selective beta-2 adrenergic agonist, via continuous intravenous infusion for five days. Prophylactic antibiotic therapy was commenced with ampicillin 1 g every 12 hours as a preventive measure against group B streptococcus (GBS) sepsis as colonization status was unknown due to technical constraints. To enhance fetal pulmonary maturation, dexamethasone was administered in three doses of 8 mg. Furthermore, in accordance with our institutional protocol, enoxaparin 40 mg daily was prescribed for thromboprophylaxis. The patient underwent close maternal and fetal monitoring, including serial laboratory evaluations and clinical assessments.

At five days post-admission, the patient experienced persistent uterine contractions and spontaneous rupture of membranes with meconium-stained amniotic fluid. Emergency removal of the cervical cerclage facilitated the vaginal delivery of the first twin, a male infant weighing 690 g. Umbilical cord ligation was performed as high as possible within the cervix under aseptic conditions, and the placenta was retained in utero. Following the expulsion of the first fetus, uterine contractions ceased.

Tocolysis was reinstated using hexoprenaline infusion and indomethacin (twice daily). Broad-spectrum antimicrobial coverage was expanded to include ceftriaxone and metronidazole, in addition to ampicillin, in response to the presence of meconium-stained amniotic fluid.

A post-delivery ultrasonographic evaluation confirmed the viability of the second fetus, who was noted to be in a breech presentation, with a normal amniotic fluid index. Two separate placentas were also visualized.

Due to maternal Rhesus D negative status, anti-D immunoglobulin was administered within 72 hours post-delivery.

At 20 days postpartum, cervical cultures confirmed *Escherichia coli *colonization, with associated leukocytosis and elevated C-reactive protein (CRP) levels (58 mg/L with a 5 mg/L normal limit). Given these findings, antibiotic therapy was adjusted to amikacin (1 vial every 12 hours for 7 days). Tocolysis with hexoprenaline infusion was reinitiated due to recurrent uterine activity. Additionally, a second course of dexamethasone (8 mg x 3 doses) was administered to further enhance fetal lung maturation.

Following 30 days of hospitalization, a pelvic examination revealed 7 cm cervical dilation and ruptured membranes in the setting of regular contractions. Due to non-progressive labor and episodes of fetal distress, characterized by persistent bradycardia, an emergency cesarean section was performed. A female neonate was delivered at 28 weeks of gestation, weighing 1,200 g, with an Apgar score of 8.

Intraoperatively, two placentas were extracted. One placenta was small, fibrotic, and calcified, with a narrow necrotic umbilical cord, while the second exhibited a retroplacental hematoma.

Postoperatively, the mother received a second dose of anti-D immunoglobulin within 72 hours and underwent a 5-day course of antimicrobial prophylaxis with cefuroxime (1.5 g every 12 hours), metronidazole (1 g every 12 hours), and gentamicin (1 vial every 12 hours). Thromboprophylaxis was continued with enoxaparin 40 mg daily, and iron supplementation (Venofer infusion, Vifor (International) Inc., Glattbrugg, Switzerland) was initiated due to anemia (hemoglobin: 11.4 g/dL).

The postoperative course was uneventful, and the patient was discharged in stable condition on postpartum day 42.

The most relevant maternal blood tests, performed at admission, 20 days postpartum, and before discharge, are presented in Table 1.

**Table 1:** Maternal blood test results at hospital admission, 20 days postpartum and at discharge. CRP: C-Reactive Protein; APTT: Activated Partial Thromboplastin Time; PT: Prothrombin Time; INR: International Normalised Ratio

Parameter	Normal Range	Hospital Admission	20 Days Postpartum	At Discharge
WBC (x10³/mm³)	4-10	35.8	24.1	9.6
Hemoglobin (g/dL)	12-16	11.1	11.4	13.5
Platelets (x10³/mm³)	150-400	207	255	296
CRP (mg/L)	<5	32	58	0.2
APTT (sec)	25-35	32.1	34.9	32.7
PT (sec)	10-13	10.6	11.6	10.7
INR	0.8-1.2	0.99	1.07	1
Fibrinogen (mg/dL)	200-400	424	573	500

Neonatal Outcomes

**Figure 1:**
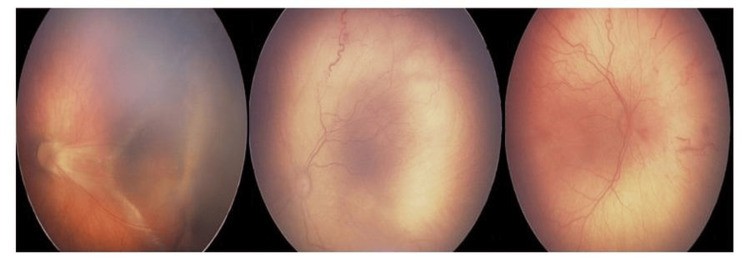
Aggressive retinopathy of prematurity in twin 1, with partial retinal detachment. Sequential fundus photographs illustrating stages of retinopathy of prematurity in the first twin. The left image demonstrates partial retinal detachment; the middle and right images show peripheral retinal vascular abnormalities and neovascularization consistent with aggressive posterior retinopathy of prematurity. These findings guided the decision for laser photocoagulation.

The first neonate, a male infant born at 24 weeks of gestation, was discharged at 37 weeks corrected gestational age. He developed mild bronchopulmonary dysplasia, intraventricular hemorrhage (resolving), and aggressive retinopathy of prematurity with partial retinal detachment, that required laser photocoagulation (Figure 1). Despite these complications, his neurological prognosis was favorable. Initial respiratory support included mechanical ventilation at birth, surfactant administration, extubation after 10 days, and non-invasive continuous positive airway pressure for an additional 10 days. Due to elevated inflammatory markers and maternal *E. coli *colonization, he received broad-spectrum antibiotic therapy for 14 days, with a favorable clinical course. Enteral feeding was initiated within the first 24 hours and was well tolerated. Given persistent oxygen dependency beyond 28 days, he underwent DART (Dexamethasone: A Randomized Trial) protocol (descending doses of dexamethasone over 10 days) for chronic lung disease.

The second neonate, a female infant born at 28 weeks, was discharged at 37 weeks of gestation, with a favorable long-term prognosis. She required only supplemental oxygen and did not necessitate surfactant therapy. Enteral nutrition was initially delayed due to abdominal distension and bilious gastric residual, but oral feeding commenced at 2 weeks of age. She exhibited intraventricular hemorrhage and aggressive retinopathy of prematurity, also requiring laser photocoagulation, with a positive outcome.

Second case

A 52-year-old multiparous woman (gravida IV, para III, abortus I) was admitted at 25 weeks of gestation with a dichorionic-diamniotic twin pregnancy, following in vitro fertilization with donor oocytes. She presented with premature rupture of membranes of the leading twin, associated with vaginal bleeding and frequent uterine contractions. Her pregnancy was closely monitored at another obstetric clinic, and she had no relevant medical history, no known chronic illnesses, or obstetric complications during the current pregnancy.

On pelvic examination, ruptured membranes and complete cervical dilation were observed. A female neonate was delivered in a cephalic presentation, weighing 730 g, with Apgar scores of 1 at 1 minute, 2 at 5 minutes, and 3 at 10 minutes. With maternal consent, the umbilical cord of the first twin was clamped and retained intrauterinely under aseptic conditions, while the placenta was left in situ. The amniotic sac of the second twin remained intact. Ultrasonographic evaluation confirmed a viable second fetus, in pelvic presentation, demonstrating normal fetal movements and reassuring Doppler parameters.

The patient was admitted to strict inpatient monitoring and initiated on tocolytic therapy, antibiotic prophylaxis, and corticosteroids. Prophylactic tocolysis was initiated using drotaverine infusion for five days, and indomethacin was given twice daily. Broad-spectrum antibiotic prophylaxis with intravenous cefuroxime (1 g every 12 hours for 5 days) was commenced to reduce the risk of intrauterine infection and maternal sepsis. To promote fetal lung maturity, dexamethasone (three doses of 8 mg intramuscularly) was administered. The maternal condition was closely monitored through clinical assessments and laboratory evaluations.

One day post-delivery of the first twin, laboratory investigations revealed elevated inflammatory markers suggestive of chorioamnionitis, including leukocytosis, C-reactive protein (CRP) elevation (23.4 mg/L, with a 5 mg/L normal limit), and hepatic dysfunction with elevated transaminase levels (aspartate aminotransferase (AST): 171 U/L, alanine aminotransferase (ALT): 158 U/L). Additionally, the patient developed anemia (Hb: 10.4 g/dL). Renal function tests and urinalysis were within normal limits. Screening for congenital intrauterine infections and sexually transmitted diseases was performed. Supportive therapy was initiated, including oral ursodeoxycholic acid 1 g/day for hepatoprotection and oral iron supplementation (100 mg once daily for 5 days) to correct anemia.

At five days post-initial delivery, the patient developed frequent contractions and active vaginal bleeding. A speculum examination revealed ongoing hemorrhage, while ultrasound confirmed fetal viability, with a cephalic presentation, normal fetal heart rate, and reduced amniotic fluid volume, but no evidence of placental abruption. In response to these findings, vaginal delivery of the second twin was expedited. The second female twin was delivered at 25 weeks and 5 days of gestation, five days after the first twin. She weighed 770 g and had Apgar scores of 1 at 1 minute, 2 at 5 minutes, and 2 at 10 minutes. The placentas were naturally expelled, with both appearing small, fibrotic, and calcified.

Post-delivery laboratory results demonstrated persistently elevated inflammatory markers, with AST: 167 U/L, ALT: 161 U/L, and C-reactive protein (CRP) rising to 203 mg/L. The platelet count remained stable (284,000/mm³), but hemoglobin continued to decline (6.3 g/dL). Empiric broad-spectrum antibiotic therapy was escalated to intravenous Clindamycin (every 8 hours) and Gentamicin (every 8 hours) for 5 days due to suspected intrauterine infection. To prevent thromboembolic events, Enoxaparin 0.4 mL subcutaneously was administered once daily.

Given the worsening anemia, a multidisciplinary team decision was made to proceed with a blood transfusion. Transvaginal ultrasound identified intrauterine blood clots and retained trophoblastic tissue (25.8 mm in the cervical isthmus). On postpartum day six, transaminase levels normalized, and CRP showed a declining trend. However, hemoglobin levels further declined to 6.1 g/dL, necessitating a second blood transfusion. Uterine evacuation via curettage was performed with maternal consent.

Maternal laboratory parameters, including inflammatory markers, liver enzymes, renal function, and coagulation profile, are summarized in Table 2, illustrating the progression of systemic inflammation and subsequent normalization following supportive treatment.

**Table 2:** Maternal blood test results at 1, 5, and 6 days postpartum and at discharge. CRP: C-Reactive Protein; APTT: Activated Partial Thromboplastin Time; PT: Prothrombin Time; INR: International Normalised Ratio; AST: Aspartate Aminotransferase; ALT: Alanine Aminotransferase

Parameter	Normal Range	1 Day Postpartum	5 Days Postpartum	6 Days Postpartum	At Discharge
WBC (x10³/mm³)	4-10	5.9	9	6	4.2
Hemoglobin (g/dL)	12-16	10.4	6.3	6.1	8.1
Platelets (x10³/mm³)	150-400	239	284	440	431
CRP (mg/L)	<5	23.41	203.51	17	4.74
AST (U/L)	10-40	171	167	23	15
ALT (U/L)	7-56	158	161	37	24
Urea (mg/dL)	7-20	20.76	39.18	21.68	15.29
Creatinine (mg/dL)	0.3-1	0.62	1.56	0.79	0.73
APTT (sec)	25-35	26.9	31	30.1	34.2
PT (sec)	10-13	12.5	14	12.6	12.3
INR	0.8-1.2	0.99	1.12	1	0.98
Fibrinogen (mg/dL)	200-400	356	637	379	336

Antibiotic therapy was discontinued after 10 days, and the patient was initiated on anticoagulation and analgesic therapy. The postpartum course was uneventful, and the patient was discharged in stable condition on postpartum day 12.

Neonatal Outcomes

The first neonate, a female infant born at 25 weeks of gestation, was discharged at 37 weeks corrected gestational age, demonstrating normal growth and neurodevelopmental outcomes. She developed respiratory distress syndrome, requiring surfactant administration and mechanical ventilation for 30 days. On day 28 of life, she underwent DART protocol (descending doses of dexamethasone for 10 days) for evolving bronchopulmonary dysplasia. Given the presence of positive non-central cultures for *Enterococcus faecium *and a positive blood culture for *Staphylococcus haemolyticus*, she was managed with broad-spectrum antibiotics. Enteral nutrition was initiated within the first 24 hours and was well tolerated. Ophthalmologic evaluation revealed mild retinopathy of prematurity that did not require intervention.

**Figure 2:**
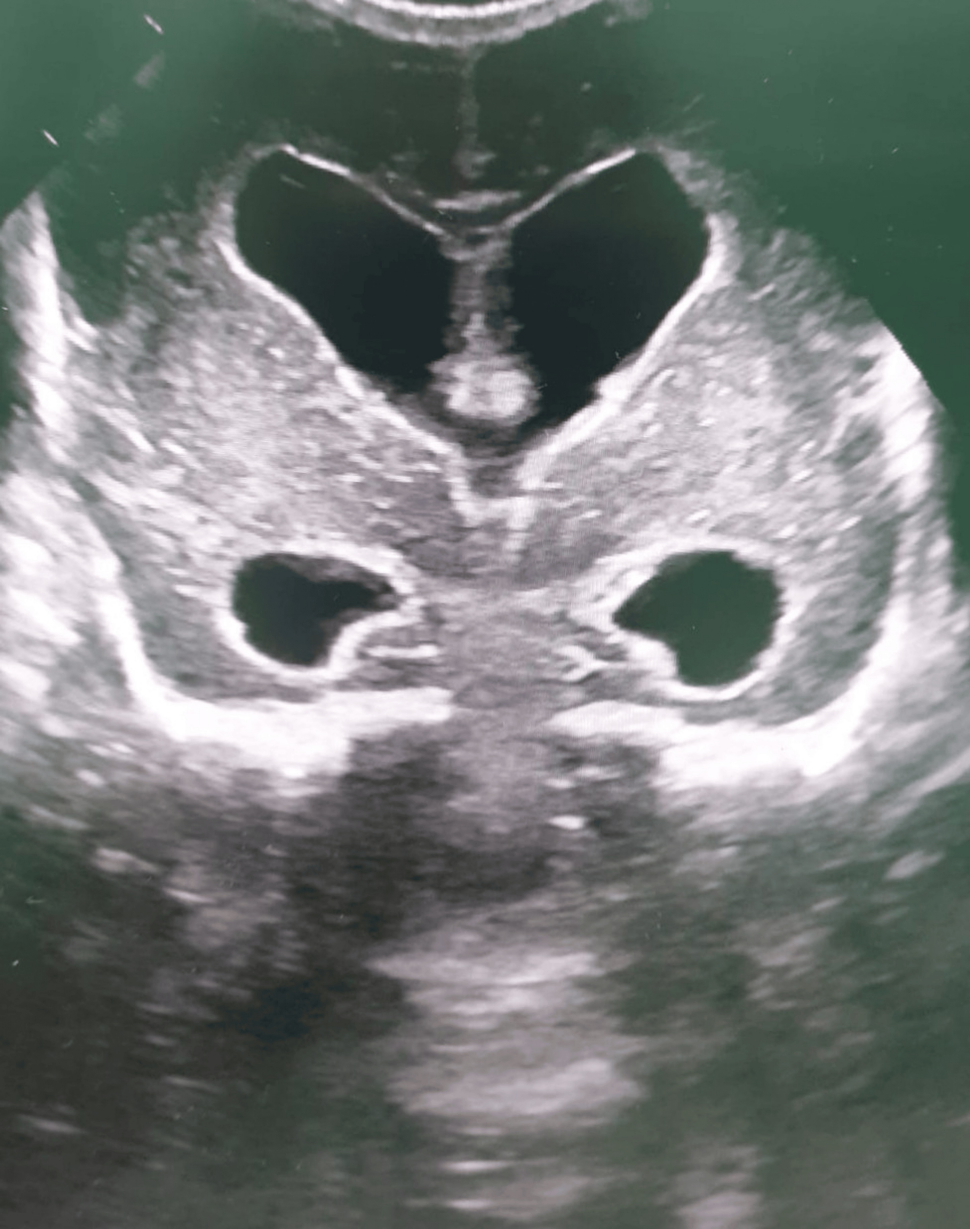
Cranial ultrasound of twin 2 demonstrating severe intraventricular hemorrhage with ventriculomegaly.

The second neonate, a female infant born at 25 weeks and 5 days, was discharged at 35 weeks corrected gestational age, with a poor neurological prognosis due to post-hemorrhagic hydrocephalus, requiring ventriculoperitoneal shunting (Figure 2). She presented with metabolic acidosis at birth, requiring immediate resuscitation. She was intubated in the delivery room, placed on mechanical ventilation, and administered surfactant upon admission to the neonatal intensive care unit. At two hours of life, she experienced cardiorespiratory arrest and required prolonged resuscitative measures. She subsequently developed severe intraventricular hemorrhage (grade IV) within the first 24 hours of life. Extubation was achieved after 27 days, without the need for corticosteroid therapy for chronic lung disease. She was administered broad-spectrum antibiotics, mirroring her sister’s treatment regimen, and demonstrated adequate enteral tolerance. However, enteral feeding was delayed until day five due to gastrointestinal immaturity.

## Discussion

Over the last decade, there has been a notable increase in research on delayed-interval delivery in multiple pregnancies. Evidence suggests that advancements in obstetric and neonatal care have led to improved success rates, with favorable perinatal outcomes in carefully selected patients. In a systematic review, Wittmann et al. reported a survival rate of 84% among retained twins in cases of delayed-interval delivery, based on 21 cases [9]. Similarly, Kalchbrenner et al. documented a survival rate of 78% in a separate cohort [10].

In our study, the inter-delivery interval ranged from 5 to 30 days, representing one of the longest durations of delayed-interval twin delivery observed in IVF pregnancies. It is well documented that for neonates born between 23 and 25 weeks of gestation, the mortality rate approaches 32%, and each additional day of gestation prolongation is associated with a 3% increase in neonatal survival [11]. Extending gestation in these cases can reduce neonatal morbidity, promote fetal maturation, and improve survival rates.

Despite the potential benefits, the optimal management strategy remains undefined. Tocolytic therapy, hospitalization, and antibiotic administration remain controversial, with significant variations in clinical practice.

Tocolysis in delayed-interval delivery

Several tocolytic agents are used in the management of preterm labor, including magnesium sulfate, nonsteroidal anti-inflammatory drugs - NSAIDs (e.g., indomethacin, sulindac), calcium channel blockers, and beta-adrenergic receptor agonists (e.g., terbutaline, hexoprenaline). Despite frequent clinical use, there is no standardized guideline defining the optimal tocolytic regimen for delayed twin delivery [3,12].

NSAIDs exert their tocolytic effect by inhibiting prostaglandin and cytokine synthesis, thereby reducing uterine contractility. Indomethacin, which has been in use since the 1970s, has been found to be the most effective in delaying delivery by at least 48 hours. However, its use is associated with neonatal complications, including premature ductus arteriosus closure, intracranial hemorrhage, renal dysfunction, periventricular leukomalacia, and necrotizing enterocolitis (NEC). Sulindac, an alternative NSAID, has been reported to have less severe neonatal side effects [13].

Current guidelines recommend nifedipine, a calcium channel blocker, as a first-line tocolytic agent due to its superior safety profile compared to beta-adrenergic receptor agonists and magnesium sulfate. However, maintenance nifedipine therapy has not been shown to significantly prolong gestation or improve neonatal outcomes [14].

Beta-adrenergic receptor agonists, such as terbutaline and hexoprenaline, exert tocolytic effects via beta-2 receptor activation, leading to increased intracellular cyclic adenosine monophosphate (cAMP) levels and smooth muscle relaxation. While terbutaline is more commonly used, hexoprenaline remains the preferred beta-2 adrenergic agonist in several countries [15]. In our study, hexoprenaline was the primary tocolytic agent, yielding favorable outcomes.

The role of tocolysis in delayed-interval delivery remains controversial. Some authors advocate for continued tocolysis until contractions cease [8], while others suggest tocolysis should be maintained even in the absence of active uterine contractility, as implemented in our cases. In both cases, hexoprenaline was continued between deliveries, regardless of uterine activity, and indomethacin was later introduced at a low dose.

Cervical cerclage and delayed twin delivery

The role of cervical cerclage in delayed twin delivery remains unclear. While cerclage is often used in singleton pregnancies to prevent preterm birth, its benefit in delayed-interval twin delivery is debated. Some studies suggest that reinforcing cervical competence after the first twin’s delivery could help prolong latency, whereas others warn that retaining the cerclage may increase infection risk [16]. In our case, cerclage removal was necessary due to spontaneous membrane rupture, which remains the most common indication for cerclage removal in multiple pregnancies.

Risk of intrauterine infection and antibiotic therapy

One of the major maternal risks associated with delayed-interval delivery is intrauterine infection, reported in 17-52% of cases, while maternal sepsis occurs in approximately 4-22% of cases. There is strong evidence linking intrauterine infections with preterm labor, with over 25% of preterm births associated with amniotic cavity infections. Proinflammatory cytokines, such as interleukin-1-beta and tumor necrosis factor-alpha, have been identified as key mediators in preterm birth pathophysiology [17].

We implemented broad-spectrum antibiotic prophylaxis, consistent with standard management protocols for premature rupture of membranes. However, there remains no consensus on the ideal antibiotic regimen, duration, or route of administration for delayed-interval twin delivery.

Long-term neonatal and neurological outcomes

While survival rates have improved, long-term neurological outcomes in delayed-interval twins remain variable. One of our neonates developed post-hemorrhagic hydrocephalus, requiring ventriculoperitoneal shunting, a well-recognized complication of extreme prematurity. Current evidence suggests that neonatal outcomes are influenced by gestational age, perinatal infection, and placental function [18]. Given the association of ART with neurodevelopmental risks, further research should investigate the cognitive and motor development of delayed-interval twins. Early functional assessments such as the General Movements Assessment (GMA) have shown promise in predicting later neurological outcomes and may be valuable in this population [19,20].

## Conclusions

This study adds valuable clinical insight into the management of delayed-interval twin delivery, particularly in IVF pregnancies. While prolonging gestation can improve neonatal survival, it is not without risks, particularly in terms of maternal infections, placental dysfunction, and long-term neurodevelopmental outcomes. The absence of standardized management guidelines highlights the need for prospective studies to define optimal tocolytic regimens, antibiotic strategies, and long-term neonatal monitoring protocols.

## References

[1] Roman AS, Fishman S, Fox N, Klauser C, Saltzman D, Rebarber A (2011). Maternal and neonatal outcomes after delayed-interval delivery of multifetal pregnancies. Am J Perinatol.

[2] Zhang J, Hamilton B, Martin J, Trumble A (2004). Delayed interval delivery and infant survival: a population-based study. Am J Obstet Gynecol.

[3] Benito Vielba M, De Bonrostro Torralba C, Pallares Arnal V, Herrero Serrano R, Tejero Cabrejas EL, Campillos Maza JM (2019). Delayed-interval delivery in twin pregnancies: report of three cases and literature review. J Matern Fetal Neonatal Med.

[4] Zorilă GL, Marinaş MC, Florea M (2016). Stillbirth in dichorionic twins discordant for major and minor anomaly, followed by asynchronous delivery - a rare occurrence. Case presentation. Rom J Morphol Embryol.

[5] Raposo MI, Cardoso M, Ormonde M, Stokreef S, Correia L, Pereira A (2017). Obstetric management of delayed-interval delivery. Case Rep Womens Health.

[6] Yang Y, Mai Z, Chen B, He F (2023). Delayed-interval delivery in twin pregnancies: 12 years' experience in one perinatal center. Int J Gynaecol Obstet.

[7] de Frias CA, Queirós AS, Simões HT (2020). Delayed-interval delivery in dichorionic twin pregnancies: a case report of 154 latency days. Rev Bras Ginecol Obstet.

[8] Cozzolino M, Seravalli V, Masini G, Pasquini L, Di Tommaso M (2015). Delayed-interval delivery in dichorionic twin pregnancies: a single-center experience. Ochsner J.

[9] Wittmann BK, Farquharson D, Wong GP, Baldwin V, Wadsworth LD, Elit L (1992). Delayed delivery of second twin: report of four cases and review of the literature. Obstet Gynecol.

[10] Kalchbrenner MA, Weisenborn EJ, Chyu JK, Kaufman HK, Losure TA (1998). Delayed delivery of multiple gestations: maternal and neonatal outcomes. Am J Obstet Gynecol.

[11] Yodoshi T, Tipton E, Rouse CA (2015). A case of delayed interval delivery with a successful hospital move. Case Rep Pediatr.

[12] Haas DM, Caldwell DM, Kirkpatrick P, McIntosh JJ, Welton NJ (2012). Tocolytic therapy for preterm delivery: systematic review and network meta-analysis. BMJ.

[13] Amin SB, Sinkin RA, Glantz JC (2007). Metaanalysis of the effect of antenatal indomethacin on neonatal outcomes. Am J Obstet Gynecol.

[14] Conde-Agudelo A, Romero R, Kusanovic JP (2011). Nifedipine in the management of preterm labor: a systematic review and metaanalysis. Am J Obstet Gynecol.

[15] (2025). WHO recommendation on tocolytic therapy for improving preterm birth outcomes. https://www.who.int/publications/i/item/9789240057227.

[16] Zhu J, Huang Y, Zeng H, Huang J, Zhang W (2024). Pregnancy outcomes of twin pregnancies with cervical insufficiency undergoing cervical cerclage. Sci Rep.

[17] Gomez-Lopez N, Romero R, Galaz J (2019). Cellular immune responses in amniotic fluid of women with preterm labor and intra-amniotic infection or intra-amniotic inflammation. Am J Reprod Immunol.

[18] Choi ES, Jung YM, Cho KD (2023). Long-term adverse neurodevelopmental outcomes of discordant twins delivered at term: a nationwide population-based study. BJOG.

[19] Toma AI, Dima V, Alexe A (2023). Correlations between head ultrasounds performed at term-equivalent age in premature neonates and general movements neurologic examination patterns. Life (Basel).

[20] Toma AI, Dima V, Rusu L (2024). Cerebral ultrasound at term-equivalent age: correlations with neuro-motor outcomes at 12-24 months corrected age. Children (Basel).

